# Translocation of aberrant left subclavian artery and resection of Kommerell’s diverticulum by total arch replacement via median sternotomy

**DOI:** 10.1186/s44215-025-00199-1

**Published:** 2025-03-06

**Authors:** Koki Yokawa, Taku Nakagawa, Makoto Kusakizako, Yosuke Tanaka, Tomonori Higuma, Kazunori Yoshida, Yoshihiro Oshima, Hidefumi Obo, Hidetaka Wakiyama

**Affiliations:** Department of Cardiovascular Surgery, Kakogawa Central City Hospital, Hyogo, Japan

**Keywords:** Kommerell’s diverticulum, Aberrant left subclavian artery, Right-sided aortic arch

## Abstract

**Background:**

Various methods for reconstructing the left subclavian artery and approaches to treat aortic aneurysms associated with Kommerell’s diverticulum and an aberrant left subclavian artery arising from a right-sided aortic arch have been reported.

**Case presentation:**

The case involved a 69-year-old woman, in whom a right-sided aortic arch with Kommerell’s diverticulum and a left subclavian artery originating from the diverticulum were incidentally observed. Severe stenosis was noted on the origin of the left subclavian artery, and the diameter of Kommerell’s diverticulum had expanded to 64 mm, resulting in dysphagia. Therefore, a total arch replacement was performed via median sternotomy. For reconstruction, the left subclavian artery was anastomosed to the left common carotid artery. Kommerell’s diverticulum was successfully resected through a median sternotomy. The postoperative course was uneventful, and the patient was discharged home without complications.

**Conclusion:**

Translocation of the aberrant left subclavian artery is a simple procedure and is effective during total arch replacement via a median sternotomy.

**Supplementary Information:**

The online version contains supplementary material available at 10.1186/s44215-025-00199-1.

## Background

Kommerell’s diverticulum is a rare congenital vascular anomaly with incidence rates ranging from 0.05 to 0.1% [1]. Transverse aortic aneurysms arch, particularly those involving the mid to the distal arch, are technically challenging to repair with conventional open techniques. The results with a combined open/endovascular approach (“hybrid repair”) in such patients were reported previously [2]. However, in cases with diverticulum-related symptoms, particularly dysphagia, the symptoms may not improve unless the diverticulum is resected. In addition, for the subclavian artery with an abnormal origin, a bypass procedure using a synthetic graft may be necessary in certain cases.

## Case presentation

**Fig. 1:**
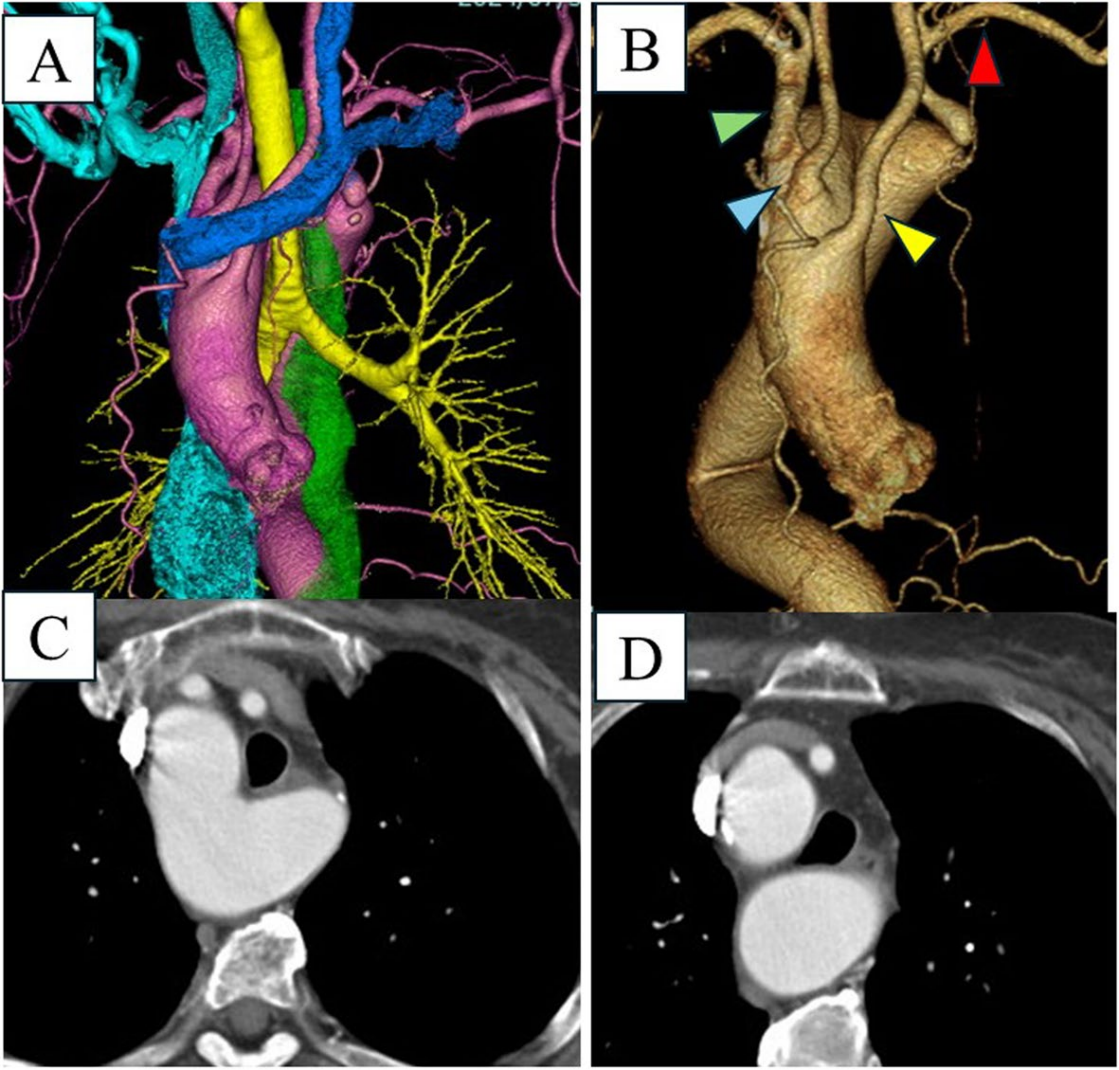
Preoperative computed tomography image. **A** Three-dimension computed tomography image. The blue structure represents the innominate vein, the yellow represents the trachea, the green represents the esophagus, and the red represents the aorta. **B** Three-dimension computed tomography image for aorta. The yellow arrowhead indicates the left common carotid artery, the blue arrowhead indicates the right common carotid artery, the green arrowhead indicates the right subclavian artery and the red arrowhead indicates the left subclavian artery. **C** Axial computed tomography image at the level of the Kommerell’s diverticulum. **D** Axial computed tomography image at the distal level of the Kommerell’s diverticulum

**Fig. 2:**
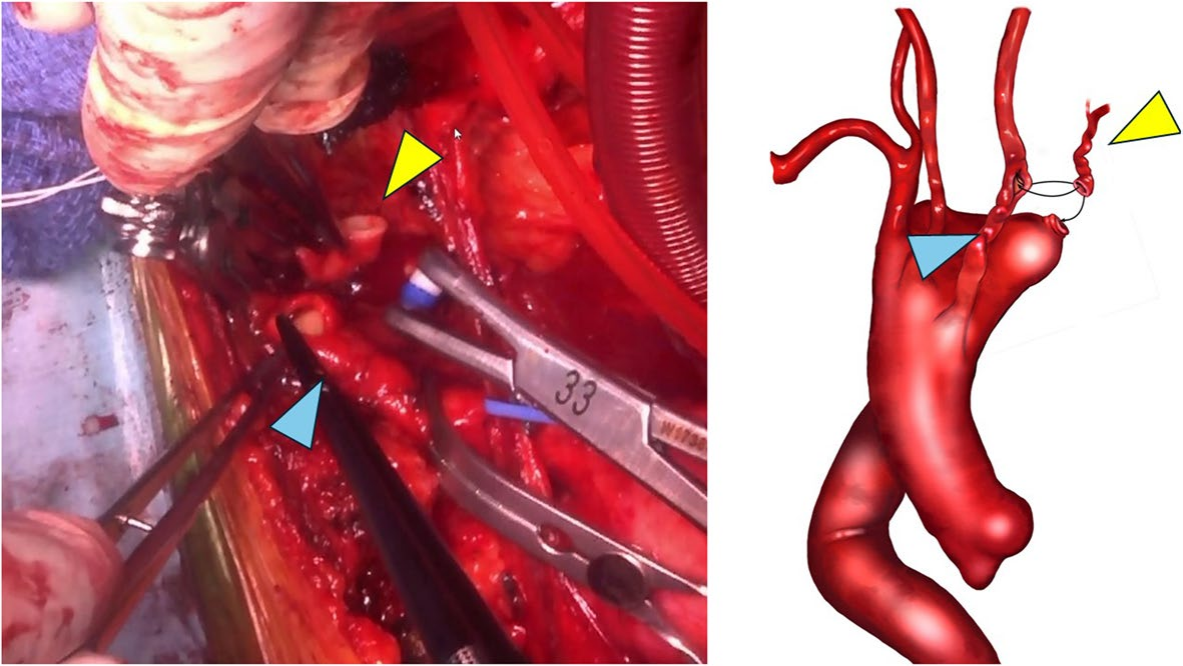
Intraoperative finding during reconstruction of the left subclavian artery. The blue arrowhead indicates the left common carotid artery and the yellow arrowhead indicates the left subclavian artery

**Fig. 3:**
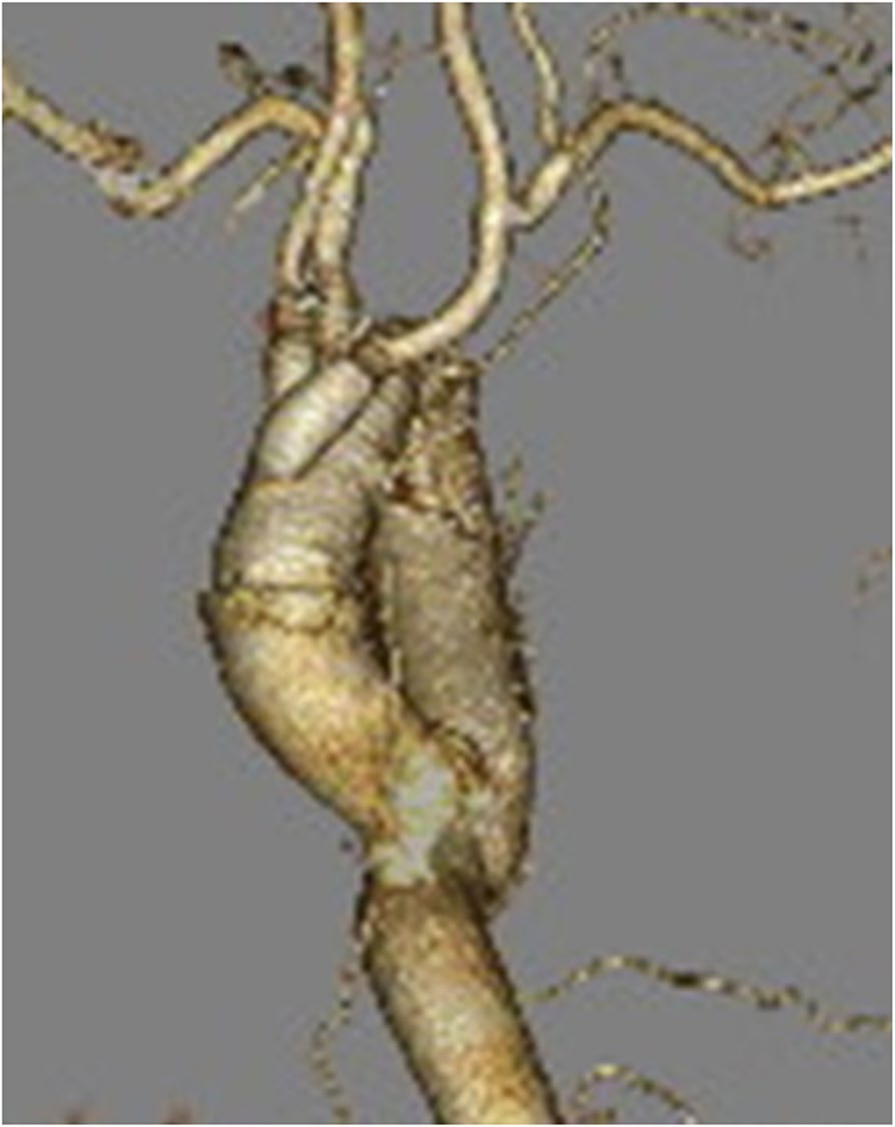
Postoperative computed tomography image

The patient was a 69-year-old woman who presented with an abnormal shadow on preoperative chest X-ray imaging conducted before an ophthalmologic procedure. Subsequent computed tomography (CT) revealed Kommerell’s diverticulum, a right-sided aortic arch, and an aberrant left subclavian artery (Fig. 1). The orifice diameter of Kommerell diameter was 27 mm and the long diameter was 62 mm. She was referred to our department for further evaluation. Notably, the left subclavian artery originated from Kommerell’s diverticulum, with significant stenosis at the origin. The patient also reported dysphagia symptoms. The systolic blood pressure in the left upper limb was approximately 30 mmHg lower than that in the right. Carotid ultrasound examination also revealed signs of retrograde flow in the left vertebral artery. Preoperative head MRI/MRA confirmed the presence of intracranial vascular communication. Esophagography revealed compression caused by the diverticulum (Supplemental Fig. S1). The presence of a dilated diverticulum and symptomatic compression indicated surgical intervention. The surgery involved performing a total aortic arch replacement via a median sternotomy alone. A left hemi-collar incision was made, partially cutting the sternocleidomastoid muscle to expose the left common carotid and left subclavian arteries. During core cooling, the left subclavian artery was end-to-side anastomosed to the left common carotid artery (Fig. 2). When the tympanic temperature reached 30 °C, the left common carotid artery was clamped, and the anastomosis started. The anastomosis time was 8 min. There was no decrease in INVOS readings before and after arterial clamping. The distal anastomosis was performed under hypothermic circulatory arrest with selective cerebral perfusion. The stepwise technique was employed for the distal anastomosis. Since a 27-mm ball sizer fit into the aorta, we selected a 26-mm four-branch J graft (Japan Lifeline, Tokyo, Japan). We transected the main graft and performed the anastomosis using the stepwise method. The postoperative course was uneventful. Dysphagia and the difference in blood pressure between the upper limbs disappeared. CT findings confirmed the patency of the left subclavian artery and the arterial wall of the Kommerell diverticulum remains intact; however, since the distal side of the aorta was transected and anastomosed, blood flow to the Kommerell diverticulum has been completely eliminated (Fig. 3).

## Discussion

Endovascular treatments and hybrid approaches have reported favorable outcomes for Kommerell’s diverticulum; however, they cannot directly relieve symptoms caused by the diverticulum, such as dysphagia and airway obstruction. In this case, given the evident compressive and associated symptoms, diverticulum resection was selected. This patient is relatively elderly at 69 years old. Descending aortic replacement using partial cardiopulmonary bypass (CPB) is generally considered less invasive than total arch replacement, which requires total CPB and deep hypothermic circulatory arrest. However, taking into account the proficiency of the surgeon and our institution in thoracotomy procedures, the ease of handling unexpected situations, and the greater familiarity of cardiovascular surgeons with median sternotomy, we ultimately determined that median sternotomy was the less invasive approach in this case. Furthermore, the fact that the patient was discharged home on postoperative day 9 supports the notion that the median sternotomy approach resulted in a lower surgical burden. While TEVAR offers a less invasive approach, the residual aneurysm would not alleviate the esophageal compression, leaving concerns for long-term complications, including the risk of an esophageal fistula. Based on these considerations, we opted for a median sternotomy, which ensures complete resection of the aneurysm and is thought to be less invasive compared to a right thoracotomy. For the reconstruction of the subclavian artery with an anatomical anomaly, bypass using a synthetic graft is a simple and commonly used method. However, in pediatric surgery, good results have been reported with translocation to the left common carotid artery [3, 4]. In this case, adding a left hemi-collar incision and partially dissecting the sternocleidomastoid muscle resulted in good visualization and reconstruction of the left common carotid and left subclavian arteries, making this approach an effective method. If intracranial left-to-right communication were hypoplastic, brain protection could still be achieved by initiating this reconstruction method at an even lower hypothermic state compared to the present case (Supplemental video S1).

In cases of a right-sided aortic arch with an aberrant left subclavian artery, the course of the left recurrent laryngeal nerve is unclear. When identifying the left subclavian artery from the mediastinal side and proceeding with dissection distally, there is a risk of unintentionally damaging the recurrent laryngeal nerve. In this case, the chosen approach was similar to that of performing a left common carotid artery-to-left subclavian artery bypass in a debranched TEVAR procedure. This approach allows for predicting the course of the recurrent laryngeal nerve, making it easier to avoid nerve damage. This approach is considered to have a low risk of injuring the recurrent laryngeal nerve.

Graft replacement for a right-sided aortic arch with Kommerell’s diverticulum is often performed under thoracotomy. However, studies have reported successful total arch replacement using only median sternotomy [5], and we consider our approach reasonable. The distal anastomosis site is not positioned at an angle directly facing the surgeon, as seen in a typical total arch replacement. Instead, the descending aorta runs towards the right and downward, making the anastomosis challenging. Even in cases of a right-sided aortic arch, if the descending aorta is located completely posterior to the trachea, it would further complicate the creation of the anastomosis site. Therefore, it is essential to confirm the positions of the esophagus, trachea, and aorta preoperatively using CT imaging. If the convergence of the Kommerell diverticulum lies caudal to the tracheal bifurcation, the anastomosis site will be positioned significantly further from the surgeon, resulting in increased difficulty. In addition, if an aortic aneurysm is formed in the distal aorta from Kommerell’s diverticulum, graft replacement under thoracotomy is recommended. Total arch replacement via median sternotomy is also deemed effective for acute aortic dissection with entry tears associated with Kommerell’s diverticulum. The stepwise technique was effective in this deep anastomosis [6].

In conclusion, in cases involving a right-sided aortic arch, Kommerell’s diverticulum, and aberrant left subclavian artery, a total arch replacement can be performed through a median sternotomy alone as usual. The left subclavian artery can be reconstructed within the same field without using a prosthetic graft by translocation.

## Supplementary Information

Supplementary Material 1: Supplemental Figure S1: Preoperative esophagography. The red arrowheads show Kommerell’s diverticulum compressing the esophagus.

Supplementary Material 2: Supplemental Video S1: Translocation of aberrant left subclavian artery.
