# Supplementary figures and images for: Translocation of aberrant left subclavian artery and resection of Kommerell’s diverticulum by total arch replacement via median sternotomy
Source: Gen Thorac Cardiovasc Surg Cases. 2025 Mar 6;4:12. doi: 10.1186/s44215-025-00199-1 (PMC11883992; doi:10.1186/s44215-025-00199-1)

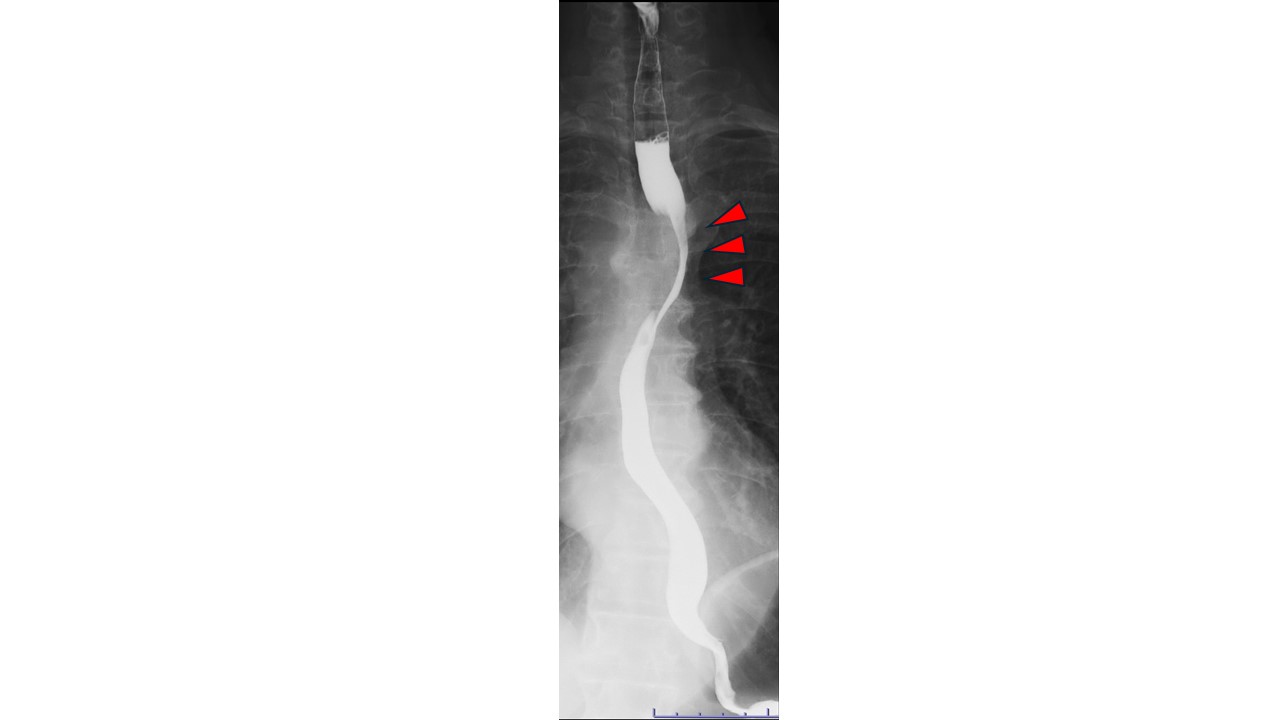

Supplement: Supplementary file 1 — Supplementary Material 1: Supplemental Figure S1: Preoperative esophagography. The red arrowheads show Kommerell’s diverticulum compressing the esophagus. [file 44215_2025_199_MOESM1_ESM.jfif]
